# Accuracy of noninvasive methods for the diagnosis of liver fibrosis in children with chronic viral hepatitis

**DOI:** 10.1186/s12876-022-02570-w

**Published:** 2022-12-09

**Authors:** A ElShahawy, MS El-Raziky, SA Sharaf, A Elsharkawy, A Enayet, H Taher

**Affiliations:** 1 Pediatrics in National Hepatology and Tropical Medicine Institiute, Cairo, Egypt; 2 Pediatrics and Pediatric Hepatology, Faculty of Medicine, Cairo University, Giza City, Egypt; 3 Chemical Pathology, Faculty of Medicine, Cairo University, Giza City, Egypt; 4 Endemic Medicine and Hepatology, Faculty of Medicine, Cairo University, Giza City, Egypt; 5 Department of Pediatrics, Kasr Alainy Medical School, Faculty of Medicine, Cairo University, Giza City, Egypt

**Keywords:** Chronic hepatitis, APRI, Hyaluronic acid, Transient elastography, Liver biopsy

## Abstract

**Background:**

Liver biopsy is the reference standard for assessing liver fibrosis. Moreover, it is an invasive procedure. Transient elastography (TE) is an accurate, noninvasive method for evaluating liver stiffness as a surrogate of liver fibrosis. The aspartate aminotransferase to platelet ratio index (APRI) and Hyaluronic acid (HA) are noninvasive alternatives to liver biopsy for detecting hepatic fibrosis. This study aimed to identify the accuracy of APRI, HA, and TE concerning liver biopsy in children with chronic viral hepatitis.

**Methods:**

This cross-sectional study included 50 children, 5–18 years with chronic viral hepatitis B (HBV) or hepatitis C (HCV) who underwent liver biopsy within nine months of laboratory tests, determining APRI & performing TE. Twenty healthy children of age and sex-matching patients were included as a control group for the serum HA levels.

**Results:**

The histopathological findings of the studied cases showed seven cases with (F0) fibrosis, 36 cases with mild (F1,2), two children with moderate (F3,4), and five children with severe (F5,6). The median (IQR) of steatosis was 4 (three had HCV). When correlating TE, APRI, and HA values in all cases with their laboratory data, there was a positive correlation between ALT and APRI values (*P*-value = 0.000), a positive correlation between AST and HA values (*P*-value = 0.02), and a negative correlation between stiffness and APRI.

The sensitivity of HA, APRI, and TE compared to fibrosis detected by histopathology was 60.5, 65.1, and 60.5%, and their specificity was 71.4, 57.1, and 85.7%, respectively. TE was significantly higher in a group with (moderate to severe) fibrosis.

**Conclusion:**

APRI, HA, and TE are good indicators of the presence of fibrosis almost with the same accuracy. TE is the only method to differentiate mild cases from those with significant fibrosis.

## Background

Liver fibrosis is a part of liver response to chronic injury through excess collagen deposition replacing normal extracellular matrix. This response to injury shows significant variations between pediatric and adult populations. In addition to the variability of the nature and the extent of some etiologies causing fibrosis in both age groups, the pediatric liver fibrosis also characterized by its unpredictable course of progression; that may be more rapid in children necessitating frequent monitoring [1]. The different inflammation and immune response and ductular reaction in pediatric fibrosis enhance the need for evaluation the application and accuracy of novel radiological and biomarker diagnostics of fibrosis [2].

Liver biopsy (LB) is the reference standard for assessing liver fibrosis. However, it is an invasive procedure that is not well accepted by patients and is burdened by complications at a low rate [3].

Transient Elastography (TE) is an accurate, noninvasive method for evaluating liver stiffness as a surrogate of liver fibrosis [4]. It is a validated method for determining liver fibrosis in adult patients with chronic hepatitis, which can reduce the use of invasive biopsies [5].

Aspartate aminotransferase to platelet ratio index (APRI) is a tool with limited expense and widespread availability; it is a promising noninvasive alternative to liver biopsy for detecting hepatic fibrosis [6].

Hyaluronic acid (HA) serum levels increase in chronic liver diseases; therefore, serum HA assessment can identify progressive liver damage early [7]. It appears to be the best individual test that reflects extracellular matrix concentration [8]. HA is a reliable, simple, noninvasive marker for assessing liver fibrosis in hepatitis C (HCV)-infected adult patients with high sensitivity and specificity [9]. Significant higher levels of serum HA have been detected in chronic viral hepatitis patients compared to healthy controls [10].

This study aimed to identify the accuracy of fibrosis serological markers APRI, HA, and TE concerning liver biopsy in children with chronic viral hepatitis.

## Methods

This cross-sectional study included 50 children (5–18 years old) with chronic viral hepatitis, either hepatitis B (HBV) or HCV. The study group had their liver biopsy within nine months of the laboratory test, determining the APRI and performing TE. Children with fatty thorax, ascites, or a pacemaker were excluded due to technical difficulty doing TE.

Twenty healthy children aged and sex-matched to the patients were included in the study as the control group for serum HA levels.

Written informed consent was obtained from each caregiver. Patients were subjected to history taking, clinical examination, complete blood cell count, liver biochemical profile, and serum level of hyaluronic acid using ELISA (BioMerieux Mary, France). APRI was calculated using Wai’s formula, which equals ((AST/upper limit of normal)/platelet count, expressed as platelets × 10^9^/L) × 100.

Liver stiffness measurements were carried out for all patients with TE (Echosens, FibroScan 502, Paris, France) which were performed in viral hepatitis specialized treatment centers by a single experienced operator. TE is a device that consists of a 5 MHz ultrasound transducer probe mounted on the axis of a vibrator. It measures liver stiffness in a volume that approximates a cylinder of 1 cm wide and 4 cm long, between 25 and 65 mm below the skin surface. The patient laid in the dorsal decubitus with the right arm in a maximal abduction, and measurements were performed on the right lobe of the liver by placing the tip of the transducer perpendicularly on the intercostal space. The median value of ten successful acquisitions was expressed in kilopascal (kPa) and was kept as representative of liver stiffness measurements. The clinical interpretation of TE depends on two critical parameters for results to be considered reliable. First, the interquartile range (IQR), which reflects the variability of the validated measures, should not exceed 30% of the median value. Second, the success rate (the ratio of the number of successful measurements to the total number of acquisitions) should be at least 60%. Interpretation of Fibrosis was divided into four stages; from F0 (No fibrosis) to F4 (severe fibrosis) according to the predefined cut-off values, which were 7.1 kPa for F ≥ 2, 9.5 kPa for F ≥ 3, and 12.5 kPa for F = 4 [11].

Liver fibrosis was diagnosed via histopathological examination. All liver biopsy details such as naked eye examination, microscopic picture, number of portal tracts, and degree of fibrosis using the Ishak score and steatosis were retrieved from files.

### Statistical analysis

Statistical Package for Social Science (SPSS, Chicago, IL 60606–6412, USA) version 17.0 was used for data analysis. Data were collected and tabulated. Mean, standard deviation (SD), or median and interquartile range (IQR) were estimates of quantitative data, while frequency and percentage were estimates of qualitative data. Differences in clinical and biochemical characteristics were tested by Student’s *t-test* or Mann–Whitney U test for quantitative data and by the Chi-square test for categorical data. A two-sided *P*-value ≤ 0.05 was considered statistically significant.

TE, HA, and APRI were assessed in association with biopsy results via the receiver operator characteristic (ROC) curve. Biopsy was categorized into no fibrosis (F0) and fibrosis (1–6). The best cutoff was selected, from which sensitivity, specificity, positive predictive value (PPV), negative predictive value (NPV), and accuracy were calculated. Markers were grouped in pairs by multiplying their values and retested for their association with biopsy results. The area under the curve of a single feature was compared with that of two markers through a comparison of two ROC curves.

One-Way ANOVA was used to determine the difference between liver stiffness and stages of fibrosis, while Kruskal–Wallis H Test was used to determine the difference between each Hyaluronic and APRI and fibrosis stages.

Spearman correlations were used to determine the linear association of clinical/ laboratory parameters with stiffness, APRI, and HA values. Box plots were drawn to show the distribution of liver stiffness and APRI values in each fibrosis stage in the HCV patients.

## Results

The study was conducted on 50 children with chronic viral hepatitis (HBV or HCV). They were 33 males and 17 females. There were 15 cases with HBV and 35 cases with HCV. The control group included 11 females and nine males with a mean age of 13.1 ± 1.8 and 11.8 ± 3.5 years, respectively.

The liver biochemical profiles of the studied group are demonstrated in Table 1. Two cases had an elevated ALT and AST with other normal liver biochemical profiles, while three had only an elevated AST.

**Table 1:** The liver biochemical profile of the studied cases

Lab	Values of all cases	Min–max	Abn values N (%)	Values in HBV *n* = 15	Values in HCV *n* = 35
Total bilirubin[N:up to 1 mg/dl] *median (IQR)*	0.4 (0.3–0.6)	0.2 – 2.8	3 (6)	0.4 (0.3–0.7)	0.5 (0.4–0.6)
Direct bilirubin[N:up to 0.3 mg/dl] *median (IQR)*	0.1 (0.1–0.1)	0 – 0.5	1 (2)	0.1 (0.1–0.1)	0.1 (0.1–0.1)
ALT[N: up to 65U/L] median (IQR)	13 (10.8–21.5)	9 –102	1 (2)	14.5(10.8–54.5)	12 (10–21)
AST[N: up to 46 U/L] median (IQR)	15 (12–32.3)	9 – 97	4 (8)	15.5 (12–44.8)	15 (12–32)
Alkph[N: up to 455 IU/L] Mean ± SD	157 ± 53.4	39 – 285	0	173.8 ± 41.9	151.9 ± 53.6
GGT[N: up to 50 IU/L] *Median (IQR)*	19.5 (14.8–27)	7 – 66	2 (4)	21 (10.5–27.8)	19 (16–27)
Albumin[N:3.5–5 g/dl] Mean ± SD	4.4 ± 0.3	3.5 – 4.9	0	4.3 ± 0.4	4.4 ± 0.3
PT [N: 13 s] Mean ± SD	13.3 ± 0.7	11.2 -15.2	0	13.1 ± 0.9	13.4 ± 0.7
INR [N: up to 1] Mean ± SD	1.02 ± 0.05	1 – 1.2	0	1.04 ± 0.05	1.02 ± 0.05

*AST* Aspartate aminotransferase, *ALT* Alanine transaminase, *GGT* Gamma Glutamyl Transferase, *PT* Prothrombin Time, *Alkph* Alkaline phosphatase, *N* Normal, *U* unit, *L* liter

There was no significant difference in the HA levels between the patients and the control (*P* = 0.1). The median (IQR) in ng/ml of HA levels of cases was 18.5 (11.8–22.3), and controls were 12 (9–19.5).

Liver stiffness was assessed in all children through performing TE as previously described. F0 was detected in 22 (44%), F0-1 in 7 (14%), F1 in 12 (24%), F1-2 in 6 (12%), F2 in 1 (2%) and F3-F4 was detected only in 2 (4%) cases (one with HBV and the other with HCV).

The histopathological findings in the liver biopsy of the studied cases revealed 7 with (F0) fibrosis, 36 with mild (F1, 2), two with moderate (F3, 4), and five children with severe (F5, 6). The median (IQR) of steatosis was 4 (three had HCV). None of the patients had a normal pathology, i.e., no fibrosis or activity.

When correlating TE, APRI, and HA values in all cases with their laboratory data, there was a positive correlation between ALT and APRI values (*P*-value = 0.000). There was a positive correlation between AST and HA values (*P*-value = 0.02). In contrast, a negative correlation was determined between stiffness and APRI.

When comparing serum levels of HA, APRI calculation, results of liver stiffness tested by TE, and the different stages of fibrosis detected by liver biopsy, there was no statistically significant difference (Table 2).

**Table 2:** The markers and stages of fibrosis in the studied group

Fibrosis by biopsy	No (0) *N* = 7	Mild (1, 2) *N* = 36	Moderate (3,4) *N* = 2	Severe (5, 6) *N* = 5	*P*-value
TE stiffness; mean ± SD in kPa	5.3 ± 1.6	6 ± 1.2	6 ± 0.5	7.9 ± 4.3	0.1
Hyaluronic; median (IQR) in ng/ml	14 (9–23)	19.5(11.3–22)	14 (6–22)	21 (15–27)	0.6
APRI; median (IQR)	0.1 (0.07–0.1)	0.1 (0.1–0.3)	0.4 (0.3–0.5)	0.1 (0.1–0.6)	0.2

APRI = [(AST/upper limit of normal) / platelet count (10 ^9^ /l)] × 100

When categorizing patients by liver biopsy according to the degree of liver fibrosis into two groups, one group with (no to mild) fibrosis and the other with (moderate to severe) fibrosis, it was found that the liver stiffness by TE was significantly higher only in a group with (moderate to severe) fibrosis. At the same time, APRI and HA showed no significant results (Table 3).

**Table 3:** The markers of fibrosis and fibrosis stages in the studied cases

Fibrosis by biopsy	No & mild *N* = 43	Moderate &severe *N* = 7	*P*-value
TE stiffness; mean ± SD [in kPa]	5.9 ± 1.3	7.3 ± 3.6	0.05*
Hyaluronic; median (IQR) [in ng/ml]	18 (11–22)	21 (15–27)	0.6
APRI; median (IQR)	0.1 (0.1–0.2)	0.2 (0.1–0.5)	0.1

APRI = [(AST/upper limit of normal) / platelet count (10 ^9^/l)] × 100

* Significant *P* value

The sensitivity of different fibrosis markers (TE, HA, and APRI) compared to the biopsy findings in the studied group was 60.5, 60.5, and 65.1%, respectively. Moreover, the specificity of different fibrosis markers (TE, HA, and APRI) compared to the biopsy findings in the studied group was 85.7, 71.4, and 57.1%, respectively (Table 4).

**Table 4:** The sensitivity, specificity, PPV, NPV, and accuracy of different fibrosis markers in the studied group associated with the biopsy findings

	The area under the curve	Best cutoff	*P* value	Sensitivity	Specificity	PPV	NPV	Accuracy
FibroScan stiffness in kPa	0.7	5.5	0.2	60.5	85.7	96.3	26.1	64
Hyaluronic in ng/ml	0.6	16.5	0.6	60.5	71.4	92.9	22.7	62
APRI	0.6	0.105	0.4	65.1	57.1	90.3	21.1	64

APRI = [(AST/upper limit of normal) / platelet count (10 ^9^/l)] × 100

## Discussion

LB is a standard method for obtaining liver tissue for histopathological evaluation in children [12]. There are several validated noninvasive diagnostic methods for determining liver fibrosis, such as serum markers (HA, APRI) and imaging with TE [13].

HA levels in the studied children with HCV and controls had no significant statistical difference (*P* = 0.09), as the cases had a HA median ± SD of 18.5 (11.8–22.3) in ng/ml and controls had 12 (9.00–19.5). These results were consistent with Valva et al. (2011), who studied serum biomarkers in detecting fibrosis in 22 children and 22 adult HCV patients and found that HCV cases and controls had no significant statistical difference because cases had a median of 10.22 (2.67–228.2), and controls had 6.36 (0–11.03) [14].

The current study found that APRI could predict fibrosis with a sensitivity of 65.1% and specificity of 57.1% in children with chronic viral hepatitis. Hassan et al. (2014) reviewed the diagnostic techniques for hepatic fibrosis. They concluded that APRI could predict significant fibrosis with a sensitivity of 72.7% and specificity of 62.4% in patients with chronic hepatitis C [15]. Nevertheless, this was consistent with Behairy et al. (2021). The latter had a comparative study between liver biopsy and noninvasive biomarkers in the assessment of hepatic fibrosis in 200 children with chronic liver diseases (HBV, HCV, AIH, and metabolic liver diseases), with 15% of their cases having chronic viral hepatitis (10% with HCV and 5% with HBV) and concluded that APRI was an excellent noninvasive alternative to liver biopsy in the detection of liver fibrosis and its extent in children with chronic liver diseases of different etiologies [16].

All studied children were examined by TE. Consistent with the current findings, the study by Mogahed et al. (2021) determined the improvement in liver stiffness in 23 pediatric HCV patients after treatment with direct-acting antivirals and found that the minority of their cases had advanced fibrosis at the baseline before treatment; F3-4 in 8% and F4 in 8% [17]. The minority of the current cases had advanced fibrosis (F3-4 in only 2%).

From this study, we can state that TE is a reliable method for the assessment of hepatic fibrosis in comparison to liver biopsy in children with chronic viral hepatitis, with a sensitivity of 60.5% and specificity of 85.7%. The accuracy of TE in comparison to LB was 64%, with PPV above 90%. TE was significantly higher (*P* = 0.05) in the group with moderate (F3, 4) to severe (F5, 6) fibrosis than in the group with no (F0) to mild (F1, 2) fibrosis based on comparing the markers of fibrosis (TE, APRI, and HA) in cases with no or mild fibrosis to moderate and severe fibrosis resulting in stiffness. That agreed with Behairy et al. (2016), who studied TE compared to LB in pediatric CLD (HBV, HCV, AIH, and metabolic liver diseases) and found stiffness correlated significantly with fibrosis. The performance of stiffness in discriminating stages of fibrosis was highly significant. Comparing the stiffness of each fibrosis stage within different etiological groups revealed a higher stiffness with higher fibrosis stages. TE outperformed HA in predicting any degree of fibrosis [18]. Similarly, Xu et al. (2021) assessed liver fibrosis by TE in 157 young children (0–6 years) with chronic HBV and found that APRI did not provide additional advantages over stiffness for determining hepatic fibrosis stages(F ≥ 2 and F ≥ 3) and concluded that TE was a promising technique for diagnosing advanced fibrosis in chronic HBV children aged 0–6 years [19]. Furthermore, Lee et al. (2013) evaluated and compared the ability of HA and TE to predict advanced hepatic fibrosis in 128 patients (pediatrics and young adults) and stated that TE outperformed HA in predicting advanced fibrosis [20].

The current study found that the accuracy of TE, HA, and APRI compared with LB was 64, 62, and 64%, respectively, with a confidence interval of 95%. PPV of all markers was above 90%. There were no significant differences between the values of markers and different stages of fibrosis detected by LB. De Lédinghen et al. (2007) found that markers of fibrosis (APRI and TE) and the histological fibrosis score were significantly correlated [21]. That disagreed with Lee et al. (2013), who stated that TE was a superior noninvasive index for detecting fibrosis rather than APRI in children aged 0–6 years with HBV-related advanced fibrosis. TE outperformed any indirect measures concerning specificity, PPV, NPV, and overall accuracy correlated to advanced fibrosis [20]. In addition, the results disagreed with Xu et al. (2021), who found that APRI did not provide additional advantages over TE for discriminating hepatic fibrosis stages (F ≥ 2 and F ≥ 3) in chronic HBV children [19]. Luo et al. (2022) studied the assessment of liver fibrosis by TE and multi-parameters model in young children with chronic hepatitis B virus infection. They stated that the diagnostic value of TE was better than that of APRI in CHB children with significant liver fibrosis [22].

This study found that the markers were grouped in pairs (e.g., hyaluronic and stiffness) and retested for their association with biopsy results. There was no statistically significant difference when we compared the area under the curve of one or two markers. Similarly, Lee et al. (2013) found that the combination of TE and HA was not better than TE alone for predicting advanced fibrosis (*P*= 0.15) [20].

The limitation of the present study was the relatively small number. However, its strength was that the TE was performed by a single experienced operator blinded to liver pathology results, and the fibrosis markers were compared to the gold standard technique of fibrosis detection.

## Conclusion

APRI, HA, and TE are excellent indicators of fibrosis, nearly with the same accuracy. In addition, TE is the only method to distinguish cases with mild from those with significant fibrosis.

## Data Availability

The datasets used and/or analysed during the current study are available from the corresponding author on reasonable request.

## Abbreviations

ALT: Alanine aminotransferase
APRI: Aspartate aminotransferase to platelet ratio index
AST: Aspartate aminotransferase
CLD: Chronic liver disease
HA: Hyalouronic acid
HBV: Hepatitis B Virus
HCV: Hepatitis C virus
IQR: Interquartile range
LB: Liver biopsy
NPV: Negative predictive value
PPV: Positive predictive value
ROC: Receiver operator characteristic
SD: Standard deviation
TE: Transient Elastography
